# Timing of hospital admission at first childbirth: A prospective cohort study

**DOI:** 10.1371/journal.pone.0281707

**Published:** 2023-02-16

**Authors:** Kristen H. Kjerulff, Laura B. Attanasio, Jennifer Vanderlaan, Kristin K. Sznajder

**Affiliations:** 1 Department of Public Health Sciences, College of Medicine, Penn State University, Hershey, Pennsylvania, United States of America; 2 Department of Obstetrics and Gynecology, Penn State Milton S. Hershey Medical Center, Hershey, Pennsylvania, United States of America; 3 School of Public Health and Health Sciences, University of Massachusetts Amherst, Amherst, Massachusetts, United States of America; 4 School of Nursing, University of Nevada, Las Vegas, Nevada, United States of America; University of Palermo: Universita degli Studi di Palermo, ITALY

## Abstract

**Background and aims:**

It is difficult for women in labor to determine when best to present for hospital admission, particularly at first childbirth. While it is often recommended that women labor at home until their contractions have become regular and ≤ 5-minutes apart, little research has investigated the utility of this recommendation. This study investigated the relationship between timing of hospital admission, in terms of whether women’s labor contractions had become regular and ≤ 5-minutes apart before admission, and labor progress.

**Methods:**

This was a cohort study of 1,656 primiparous women aged 18–35 years with singleton pregnancies who began labor spontaneously at home and delivered at 52 hospitals in Pennsylvania, USA. Women who were admitted before their contractions had become regular and ≤ 5-minutes apart (early admits) were compared to those who were admitted after (later admits). Multivariable logistic regression models were used to assess associations between timing of hospital admission and active labor status on admission (cervical dilation 6–10 cm), oxytocin augmentation, epidural analgesia and cesarean birth.

**Results:**

Nearly two-thirds of the participants (65.3%) were later admits. These women had labored for a longer time period before admission (median, interquartile range [IQR] 5 hours (3–12 hours)) than the early admits (median, (IQR) 2 hours (1–8 hours), p < 0.001); were more likely to be in active labor on admission (adjusted OR [aOR] 3.78, 95% CI 2.47–5.81); and were less likely to experience labor augmentation with oxytocin (aOR 0.44, 95% CI 0.35–0.55); epidural analgesia (aOR 0.52, 95% CI 0.38–0.72); and cesarean birth (aOR 0.66, 95% CI 0.50–0.88).

**Conclusions:**

Among primiparous women, those who labor at home until their contractions have become regular and ≤ 5-minutes apart are more likely to be in active labor on hospital admission and less likely to experience oxytocin augmentation, epidural analgesia and cesarean birth.

## Introduction

Towards the end of pregnancy women begin to experience uterine contractions which gradually increase in frequency and intensity as the body prepares for childbirth. This process begins with early labor and eventually transitions to active labor, variously defined as beginning at cervical dilation of 4 to 6 centimeters [1,2]. Women often want to go to the hospital when they are in early labor in hopes of finding a safe and secure environment in which to labor [3] and supportive care provided by healthcare professionals [4]. However, women with low-risk pregnancies are often encouraged to labor at home until they are in active labor [4–6]. This is because a substantial body of evidence indicates that women who are admitted in early labor are more likely to experience intrapartum interventions, including labor augmentation with oxytocin, epidural analgesia and cesarean birth [1,7–12].

There have been several randomized, controlled trials (RCTs) to investigate outcomes of programs designed to delay admission to labor wards until active labor has begun [13,14]. In a Cochrane review of five RCT’s the authors concluded that: 1.) It is not clear if these programs reduce the rate of cesarean or instrumental birth, and 2.) Some evidence indicates that they may reduce labor augmentation with oxytocin and receipt of epidural analgesia, and increase maternal satisfaction [14]. However, hospital policies and programs to delay admission until active labor may leave women and their families feeling abandoned and unsupported at a time when they are most vulnerable [15,16]. Several studies have reported that being refused admission and sent home can be frightening and upsetting for some women in labor and their families [17–19]. In addition, there is also the risk that labor will progress quickly and the woman who has been sent home may give birth in less-than-ideal circumstances, such as while being transported [20]. The World Health Organization (WHO) advises that “for healthy pregnant women presenting in spontaneous labour, a policy of delaying labour ward admission until active first stage is recommended only in the context of rigorous research” [21].

Women who attend structured antenatal education programs, such as the ‘Ready for Child’ program in Denmark [22], have been found to be less worried about medical and birth issues in late pregnancy [23], more likely to arrive at the maternity ward in active labor, and use less epidural analgesia during labor [24]. However, many women in the U.S. do not attend antenatal education classes before childbirth [25] or attend only one or two brief classes [26]. Additionally, women who are less educated and lower income are less likely to attend antenatal education classes [27].

Pregnant women are increasingly turning to the internet for answers to their pregnancy and childbirth questions [28,29]. In order to help women in labor determine whether or not they are in active labor, hospitals often provide recommendations on their websites as to when women in labor should present for hospital admission. In response to a search engine query “When should a woman in labor go to the hospital?”, we found the following recommendations: “When you have regular, painful contractions lasting one minute each and occurring at least every five minutes for more than two hours” [30], “You are having regular contractions, every four minutes, lasting one minute, happening for at least one hour” [31], “When your contractions feel strong to you, last 45 to 60 seconds each and occur every 3 to 4 minutes for at least 2 hours” [32], and “For a first delivery, you are having regular contractions every 5-minutes or less for one hour”[33]. Women are often given similar recommendations in childbirth education classes and in visits with clinicians during their last weeks of pregnancy. However, we found little research that investigated the relationship between timing of hospital admission, in terms of whether women’s labor contractions had become regular and ≤ 5-minutes apart before admission, and labor progress after hospital admission. Therefore, the primary aims of this study were to: 1.) Measure the percent of women whose contractions had become regular and ≤ 5-minutes apart before hospital admission among primiparous women with singleton pregnancies who began labor at home, and 2.) Investigate the association between timing of hospital admission and six outcomes—admission in active labor (≥ 6 centimeters cervical dilation), labor augmentation with oxytocin, receipt of epidural anesthesia, cesarean birth, maternal morbidities, and neonatal morbidities, controlling for relevant confounding variables. A secondary aim was to evaluate associations between duration of labor at home after contractions had become regular and ≤ 5-minutes apart and the six study outcomes.

## Materials and methods

### Study design and inclusion criteria

This was a secondary data analysis study, using data from the First Baby Study (FBS) [34,35]. The FBS, was an observational prospective cohort study, designed to investigate the association between mode of first birth and subsequent childbearing. Information about the design of the FBS, sample size and power calculations, recruitment and enrollment, and sample representativeness have been reported previously [34,35]. This paper followed the Strengthening the Reporting of Observational Studies in Epidemiology (STROBE) guidelines. The S1 Checklist can be seen in Supporting Information.

FBS participants were recruited through childbirth education classes, hospital tours, targeted mailings, intra-hospital postings, posters, flyers and newspaper ads throughout the state of Pennsylvania. Women were eligible to participate in the FBS if they were pregnant, expecting their first child, aged 18–35 years old, with a singleton pregnancy, English or Spanish speaking, and planning to deliver in a hospital in Pennsylvania. Women were not eligible if they were planning to deliver at home or in a birthing center not affiliated with a hospital, planning for the child to be adopted, planning to have a tubal ligation at the time of birth, or if they had a prior pregnancy of 20 weeks gestation or longer. Women who delivered before 34 weeks gestation were also excluded. These inclusion and exclusion criteria were chosen based on the primary goal of the FBS—to study the association between mode of first birth (vaginal or cesarean) and subsequent childbearing [34,35]. The participants delivered in 2009 to 2011 at hospitals in Pennsylvania. Investigation of the representativeness of the study sample indicated that the participants in this study were significantly more likely to be white non-Hispanic, married, and to have college degrees and private insurance than women delivering their first child in Pennsylvania as a whole, but were not different in rate of cesarean birth [34].

Interviews were conducted by telephone by trained interviewers employed by the Penn State Center for Survey Research, using a computer-aided telephone interview system. Participants were interviewed by telephone in their third trimester (baseline) and again at 1, 6, 12, 18, 24, 30 and 36 months postpartum. The birth certificate and hospital discharge data for the births were obtained and linked to the survey data.

### Measures

For this study, we used variables from the baseline and 1-month postpartum interviews, and the birth certificate and hospital discharge data. Background information about maternal age, race/ethnicity, education, and marital status were obtained as part of the baseline interview. Gestational age, pre-pregnancy body mass index (BMI) and newborn birth weight were obtained from the birth certificate data. Type of health insurance (public or private) and mode of birth were obtained from the hospital discharge data. Premature rupture of membranes was identified if reported in the birth certificate data or in the hospital discharge data. Intrapartum interventions, including labor augmentation with oxytocin and epidural anesthesia, were measured in the 1-month postpartum interview. In addition, women were asked if they had attended childbirth education classes during pregnancy and if they had received care from a midwife. We used three dichotomous indicators developed and validated by Korst et al [36] to measure childbirth morbidity, identified on the basis of the ICD-9 CM codes reported in the hospital discharge data. The first (high-risk pregnancy) was a composite measure indicating the presence of one or more pregnancy complications that would define a pregnancy as high-risk—including conditions such as antepartum bleeding, asthma, diabetes, and hypertension. The second (maternal morbidities) was a composite measure indicating the occurrence of one or more labor and birth-related complications for the mother—including conditions such as 3^rd^ or 4^th^ degree laceration, transfusion, and admission to the adult intensive care unit. The third (neonatal morbidities) was a composite measure indicating the occurrence of one or more labor and birth complications for the newborn—including conditions such as birth trauma/injuries, hypoxia/asphyxia, and admission to the neonatal intensive care unit. We also defined a low-risk subsample among our participants in order to asess how this factor was associated with admission before or after contractions had become regular and ≤ 5-minutes apart. Using criteria similar to that used in previous studies to define a low-risk sample [9,37,38], women were classified as low-risk if they had uncomplicated pregnancies (none of the pregnancy complications identified by Korst et al [36] to denote a high-risk pregnancy), vertex presentation, who did not experience premature rupture of membranes, and delivered at gestational age of ≥ 37 weeks.

Information about women’s labor were obtained from questions asked in the 1-month postpartum interview, as follows:

1. Duration of labor before hospital admission: *“Were you in labor when you arrived at the hospital*?*”* Those who answered “yes” were then asked “*How long had you been in labor when you arrived at the hospital*?*”*
2. Contractions had become regular and ≤ 5-minutes apart before admission: *“At some point did your contractions become regular and 5-minutes or less apart*?*”* Those who answered “yes” were then asked *“Was this before or after you arrived at the hospital*?”
3. Time period in which contractions were regular and ≤ 5-minutes apart before admission: Those who reported that their contractions had become regular and ≤ 5-minutes apart before admission were asked *“How long before you arrived at the hospital did your contractions become regular and 5-minutes or less apart*?*”*
4. Cervical dilation at admission: *“How many centimeters was your cervix dilated when you were first examined after you were admitted to the hospital for delivery*?*”*
5. Labor pain before receipt of analgesia: *“When you were in labor*, *how painful was it for you during contractions*, *before you received any type of pain medication*? *Use a scale of 0 to 10*, *0 being no pain and 10 being the worst pain imaginable*.*”* This 0–10 scale is commonly used to assess labor pain [39], as well as other types of clinical pain, exhibiting good reliability and validity [40].

### Data analysis

In this study, we investigated the associations between two independent variables and six dependent variables. The two independent variables were: 1.) timing of hospital admission (early admits compared to later admits) among those women who began labor spontaneously at home, and 2.) Duration of labor at home after contractions had become regular and ≤ 5-minutes apart, among the later admits. This variable was classified into three categories: ≤ 1-hour, > 1–2 hours, and > 2 hours. The six dependent variables were: 1.) admission in active labor (6–10 cm), 2.) labor augmentation with oxytocin, 3.) receipt of epidural analgesia, 4.) cesarean birth, 5.) maternal morbidities, and 6.) neonatal morbidities. Although many of the previous studies on the timing of hospital admission have used a cutoff value of 4 centimeters cervical dilation or higher to denote active labor, recent studies suggest that a threshold of 6 centimeters cervical dilation as indicative of active labor is more realistic [41,42].

Descriptive statistics were calculated for the study variables, including frequency and percent for categorical variables, and means and standard deviations for continuous variables, with the exception of the variable of overall duration of labor at home. Because this variable was highly skewed, we calculated the median and interquartile range, as is commonly done with measures of labor duration [43]. Women whose contractions had not become regular and ≤ 5-minutes apart by the time of hospital admission (early admits) were compared to those whose contractions had become regular and ≤ 5-minutes apart before hospital admission (later admits) via chi-square analyses for categorical variables, t-tests for continuous variables, and the Mann-Whitney U test for overall duration of labor at home.

Multivariable logistic regression models were used to measure the associations between the first independent variable, timing of hospital admission (early admits in comparison to later admits), and our six dependent variables. In these models we controlled for the maternal demographic factors of age, education, race/ethnicity and type of insurance coverage at birth (public or private) and factors that would likely be taken into account when advising women in labor as to when they should go to the hospital; these were the presence of one or more pregnancy complications that would denote a pregnancy as high-risk [36], overall duration of labor at home, premature rupture of membranes, pain level before receipt of analgesia, and gestational age.

Multivariable logistic regression models were used to measure the association between the second independent variable, duration of labor at home after contractions had become regular and ≤ 5-minutes apart, among the later admits (≤ 1-hour, > 1–2 hours, and > 2 hours), and the six dependent variables. For these models we controlled for the maternal demographic factors of age, education, race/ethnicity and type of insurance coverage at delivery (public or private) and factors that would likely be taken into account when advising women in labor as to when they should go to the hospital; these were the presence of one or more pregnancy complications that would define a pregnancy as high-risk [36], premature rupture of membranes, pain level before receipt of analgesia, and gestational age. We did not control for overall duration of labor at home in these six models, because it was highly colinear with the independent variable—duration of labor at home after contractions had become regular and ≤ 5-minutes apart. Associations were considered to be significant if p values were < .05. All statistical analyses were performed using SPSS version 28 statistical program (Chicago, USA).

### Ethical approval and informed consent

The FBS was approved by the Penn State College of Medicine Institutional Review Board (IRB), as well as the IRBs of the hospitals and other organizations involved with participant recruitment. Informed consent was conducted in person or by telephone and all participants provided signed, written informed consent.

## Results

There were 3,006 women who were enrolled in the FBS and completed the baseline and 1-month interviews. Women were excluded from this study of timing of hospital admission if they were induced (n = 1,017), admitted to the hospital before the onset of labor (n = 162), had a planned cesarean (n = 160), or were missing data on one or more key variables (n = 11). This left a sample size of 1,656 participants, all of whom were in labor on hospital admission. These women delivered at 52 hospitals in Pennsylvania.

The demographic and clinical data for the women in the study are included in Table 1. The study participants identified predominantly as white non-Hispanic (83.9%), most had private health insurance (78.6%), and more than half had college degrees or higher (58.5%). A third of the women (33.3%) had one or more pregnancy complications that denoted a high-risk pregnancy, and 10% had experienced premature rupture of membranes. Overall, 12.4% of the women were in active labor (cervical dilation of 6 cm or greater) at the time of hospital admission. The participants reported laboring a median (IQR) of 4 (2–10) hours before hospital admission.

**Table 1:** Maternal and infant characteristics overall and by timing of hospital admission.

		Timing of hospital admission		
Characteristics	Overall (n = 1,656) n (%)	Early admits (n = 574) n (%)	Later admits (n = 1,082) n (%)	p-value
**Maternal characteristics**				
Maternal age (years)				0.089
18–24	478 (28.9)	175 (30.5)	303 (28.0)	
25–29	701 (42.3)	222 (38.7)	479 (44.3)	
30–35	477 (28.8)	177 (30.8)	300 (27.7)	
Education				0.505
High school degree or less	244 (14.7)	90 (15.7)	154 (14.2)	
Some college or technical	443 (26.8)	159 (27.7)	284 (26.2)	
College graduate or higher	969 (58.5)	325 (56.6)	644 (59.5)	
Private insurance	1,301 (78.6)	434 (75.6)	867 (80.1)	0.033
Race/Ethnicity				0.322
White non-Hispanic	1,389 (83.9)	477 (83.1)	912 (84.3)	
Black non-Hispanic	119 (7.2)	50 (8.7)	69 (6.4)	
Hispanic	77 (4.6)	25 (4.4)	52 (4.8)	
Other	71 (4.3)	22 (3.8)	49 (4.5)	
Childbirth education class	1,222 (73.8)	399 (69.5)	823 (76.1)	0.004
Pre-pregnancy BMI (kg/m^2)^				0.233
Underweight/Normal (< 25.0)	1,041 (62.9)	346 (60.3)	695 (64.2)	
Overweight (25.0–29.9)	366 (22.1)	132 (23.0)	234 (21.6)	
Obese (30.0+)	249 (15.0)	96 (16.7)	153 (14.1)	
^a^High-risk pregnancy	552 (33.3)	233 (40.6)	319 (29.5)	< 0.001
Premature rupture of membranes	166 (10.0)	107 (18.6)	59 (5.5)	< 0.001
Cervical dilation on admission, cm (Mean, SD)	3.6 (1.9)	2.8 (1.7)	3.9 (2.0)	< 0.001
Admitted in active labor (6–10 cm cervical dilation)	206 (12.4)	28 (4.9)	178 (16.5)	< 0.001
Overall duration of labor at home, hours (Median, IQR)	4 (2–10)	2 (1–8)	5 (3–12)	< 0.001
Overall duration of labor at home > 4 hours	825 (49.8)	211 (36.8)	614 (56.7)	< 0.001
^b^Pain level before analgesia (0–10 scale) (Mean, SD)	8.1 (1.7)	7.8 (2.0)	8.2 (1.5)	< 0.001
Birth attended by midwife	435 (26.4)	126 (22.1)	309 (28.6)	0.004
^c^Maternal morbidities	447 (27.0)	164 (28.6)	283 (26.2)	0.292
**Infant characteristics**				
Gestational age (weeks)				< 0.001
Late preterm (35–36)	55 (3.3)	32 (5.6)	23 (2.1)	
Early term (37–38)	307 (18.5)	136 (23.7)	171 (15.8)	
Full term (39–40)	1,121 (67.7)	350 (61.0)	771 (71.3)	
Late/post term (41+)	173 (10.4)	56 (9.8)	117 (10.8)	
Newborn birth weight (grams)				0.248
Low (< 2,500)	38 (2.3)	18 (3.1)	20 (1.8)	
Normal (2,500–4,000)	1,489 (89.8)	511 (89.0)	978 (90.4)	
High (> 4,000)	129 (7.8)	45 (7.8)	84 (7.8)	
^d^Neonatal morbidities	211 (12.7)	81 (14.1)	130 (12.0)	0.223
^e^Low-risk subsample	995 (60.1)	272 (47.4)	723 (66.8)	< 0.001
**Obstetric interventions**				
Labor augmented via oxytocin	816 (49.3)	364 (63.4)	452 (41.8)	< 0.001
Epidural analgesia	1,397 (84.4)	515 (89.7)	882 (81.5)	< 0.001
Cesarean birth	308 (18.6)	138 (24.0)	170 (15.7)	< 0.001

Early admits: Women who were admitted before their contractions had become regular and ≤ 5-minutes apart.

Later admits: Women who were admitted after their contractions had become regular and ≤ 5-minutes apart.

^a^High-risk pregnancy: Composite measure indicating the presence of one or more pregnancy complications that would define a pregnancy as high-risk, identified by the ICD-9 CM codes reported in the hospital discharge data, as described in Korst et al [36].

^b^Pain scale: 0 = no pain, 10 = the worst pain imaginable.

^c^Maternal morbidities: Composite measure indicating the occurrence of one or more maternal labor and delivery complications, identified by the ICD-9 CM codes reported in the hospital discharge data, as described in Korst et al [36].

^d^Neonatal morbidities: Composite measure indicating the occurrence of one or more neonatal labor and delivery related complications, identified by the ICD-9 CM codes reported in the hospital discharge data, as described in Korst et al [36].

^e^Low-risk subsample: Women with uncomplicated pregnancies (none of the pregnancy complications identified by Korst et al [36] to denote a high-risk pregnancy), vertex presentation, who did not experience premature rupture of membranes and delivered at gestational age ≥ 37 weeks.

Summary statistics are median and interquartile range (IQR) for labor duration, mean ± SD for other continuous variables, and frequency (%) for categorical variables. P-values are based on chi-square analyses for categorical variables, independent samples Mann-Whitney U test for labor duration, and two-tailed t-test for the other continuous variables.

Overall, 65.3% of the women reported that their contractions had become regular and ≤ 5-minutes apart before hospital admission (later admits). We compared the early admits to later admits via bivariate analyses (Table 1). These groups were not significantly different in age, education or race/ethnicity, but the later admits were more likely to have taken childbirth education classes, to have private insurance, and to have their birth attended by a midwife. The later admits reported higher pain levels before receipt of analgesia, and had labored significantly longer before admission, (median (IQR) 5 (3–12) hours) than the early admits, who labored a median (IQR) of 2 (1–8) hours, p < 0.001. The early admits were more likely to have a high-risk pregnancy than the later admits (40.6% vs. 29.5%), to have experienced premature rupture of membranes (18.6% vs. 5.5%), and to deliver before 37 weeks gestation (5.6% vs. 2.1%). Overall, 47.4% of the early admits were-low risk, in comparison to 66.8% of the later admits (p < 0.001). Early admits had significantly lower average cervical dilation on admission (mean ± SD cm, 2.8 ± 1.7) than later admits (mean ± SD cm, 3.9 ± 2.0), and were significantly less likely to be in active labor on admission (4.9%) than later admits (16.5%), p < 0.001. Early admits were significantly more likely to experience labor augmentation with oxytocin than later admits (63.4% vs. 41.8%, p < 0.001); epidural analgesia (89.7% vs. 81.5%, p < 0.001); and cesarean birth (24.0% vs. 15.7%, p < 0.001). However, early admits were not different from later admits in the outcomes of maternal or neonatal morbidities.

In the multivariable logistic regression models to compare early admits to later admits while controlling for confounding variables (Table 2), the later admits were more likely to be in active labor on admission (adjusted OR [aOR] 3.78, 95% CI 2.47–5.81); and were less likely to experience labor augmentation with oxytocin (aOR 0.44, 95% CI 0.35–0.55); epidural analgesia (aOR 0.52, 95% CI 0.38–0.72); and cesarean birth (aOR 0.66, 95% CI 0.50–0.88).

**Table 2:** Associations between timing of hospital admission (comparing early admits to later admits) and the six study outcomes (n = 1,656).

Outcomes	Adjusted OR	95% CI	p-value
Admitted in active labor (6–10 cm cervical dilation)	3.78	2.47–5.81	< 0.001
Labor augmented via oxytocin	0.44	0.35–0.55	<0.001
Epidural analgesia	0.52	0.38–0.72	<0.001
Cesarean birth	0.66	0.50–0.88	0.004
^a^Maternal morbidities	1.05	0.82–1.34	0.713
^b^Neonatal morbidities	1.05	0.76–1.45	0.767

Logistic regression models, controlling for maternal age, education, race/ethnicity, insurance coverage, high-risk pregnancy (Korst et al [36]), premature rupture of membranes, gestational age, pain level before receipt of analgesia, and overall duration of labor at home > 4 hours.

^a^Maternal morbidities: Composite measure indicating the occurrence of one or more maternal labor and delivery complications, identified by the ICD-9 CM codes reported in the hospital discharge data, as described in Korst et al [36].

^b^Neonatal morbidities: Composite measure indicating the occurrence of one or more neonatal labor and delivery related complications, identified by the ICD-9 CM codes reported in the hospital discharge data, as described in Korst et al [36].

Table 3 reports the results of bivariate analyses to investigate the associations between duration of labor at home after contractions became regular and ≤ 5-minutes apart among the later admits, and the maternal and infant characteristics and obstetric interventions. Few of these associations were significant. It was not surprising that duration of labor at home after contractions became regular and ≤ 5-minutes apart would be associated with overall duration of labor at home, because the former is a subset of the later. The only other variable that was significantly associated with duration of labor at home after contractions became regular and ≤ 5-minutes apart was gestational age (p = 0.031), indicating that the women who labored at home after contractions became regular and ≤ 5-minutes apart for an hour or less were more likely to be late preterm or early term than the women who labored > 1–2 hours or > 2 hours. Duration of labor at home after contractions had become regular and ≤ 5-minutes apart among the later admits was not associated with any of the six study outcomes in the bivariate analyses, as shown in Table 3.

**Table 3:** Maternal and infant characteristics by duration of labor at home after contractions had become regular and ≤ 5-minutes apart (n = 1,082).

	Duration of labor at home after contractions became regular and ≤ 5-minutes apart			
Characteristics	≤ 1 hour (n = 459) n (%)	> 1–2 hours (n = 288) n (%)	> 2 hours (n = 335) n (%)	p-value
**Maternal characteristics**				
Maternal age (years)				0.296
18–24	138 (30.1)	71 (24.7)	94 (28.1)	
25–29	188 (41.0)	141 (49.0)	150 (44.8)	
30–35	133 (29.0)	76 (26.4)	91 (27.2)	
Education				0.118
High school degree or less	78 (17.0)	39 (13.5)	37 (11.0)	
Some college or technical	114 (24.8)	71 (24.7)	99 (29.6)	
College graduate or higher	267 (58.2)	178 (61.8)	199 (59.4)	
Private insurance	370 (80.6)	237 (82.3)	260 (77.6)	0.325
Race/Ethnicity				0.594
White non-Hispanic	384 (83.7)	253 (87.8)	275 (82.1)	
Black non-Hispanic	31 (6.8)	13 (4.5)	25 (7.5)	
Hispanic	24 (5.2)	11 (3.8)	17 (5.1)	
Other	20 (4.4)	11 (3.8)	18 (5.4)	
Childbirth education class	341 (74.3)	222 (77.1)	260 (77.6)	0.497
Pre-pregnancy BMI (kg/m^2)^				0.797
Underweight/Normal (< 25.0)	285 (62.1)	190 (66.0)	220 (65.7)	
Overweight (25.0–29.9)	104 (22.7)	60 (20.8)	70 (20.9)	
Obese (30.0+)	70 (15.3)	38 (13.2)	45 (13.4)	
^a^High-risk pregnancy	128 (27.9)	87 (30.2)	104 (31.0)	0.598
Premature rupture of membranes	29 (6.3)	12 (4.2)	18 (5.4)	0.451
Cervical dilation on admission, cm (Mean, SD)	3.99 (1.99)	3.91 (1.90)	3.90 (2.01)	0.810
Admitted in active labor (6–10 cm cervical dilation)	77 (16.8)	48 (16.7)	53 (15.8)	0.932
Overall duration of labor at home, hours (Median, IQR)	3.0 (1.0–8.0)	5.0 (3.0–12.0)	9.0 (5.0–16.0)	< 0.001
Overall duration of labor at home > 4 hours	183 (39.9)	170 (59.0)	261 (77.9)	< 0.001
^b^Pain level before analgesia (0–10 scale) (Mean, SD)	8.24 (1.45)	8.09 (1.61)	8.33 (1.39)	0.132
Birth attended by midwife	119 (26.0)	92 (32.1)	98 (29.3)	0.201
^c^Maternal morbidities	110 (24.0)	81 (28.1)	92 (27.5)	0.365
**Infant characteristics**				
Gestational age (weeks)				0.025
Late preterm (35–36)	16 (3.5)	3 (1.0)	4 (1.2)	
Early term (37–38)	83 (18.1)	36 (12.5)	52 (15.5)	
Full term (39–40)	307 (66.9)	223 (77.4)	241 (71.9)	
Late/post term (41+)	53 (11.5)	26 (9.0)	38 (11.3)	
Newborn birth weight (grams)				0.410
Low (< 2,500)	8 (1.7)	6 (2.1)	6 (1.8)	
Normal (2,500–4,000)	423 (92.2)	259 (89.9)	296 (88.4)	
High (> 4,000)	28 (6.1)	23 (8.0)	33 (9.9)	
^d^Neonatal morbidities	52 (11.3)	42 (14.6)	36 (10.7)	0.285
^e^Low-risk subsample	306 (66.7)	195 (67.7)	222 (66.3)	0.926
**Obstetric interventions**				
Labor augmented via oxytocin	185 (40.3)	119 (41.3)	148 (44.2)	0.541
Epidural analgesia	382 (83.2)	236 (81.9)	264 (78.8)	0.278
Cesarean birth	71 (15.5)	43 (14.9)	56 (16.7)	0.815

^a^High-risk pregnancy: Composite measure indicating the presence of one or more pregnancy complications that would define a pregnancy as high-risk, identified by the ICD-9 CM codes reported in the hospital discharge data, as described in Korst et al [36].

^b^Pain scale: 0 = no pain, 10 = the worst pain imaginable.

^c^Maternal morbidities: Composite measure indicating the occurrence of one or more maternal labor and delivery complications, identified by the ICD-9 CM codes reported in the hospital discharge data, as described in Korst et al [36].

^d^Neonatal morbidities: Composite measure indicating the occurrence of one or more neonatal labor and delivery related complications, identified by the ICD-9 CM codes reported in the hospital discharge data, as described in Korst et al [36].

^e^Low-risk subsample: Women with none of the pregnancy complications identified by Korst et al [36] to define a woman as having a high-risk pregnancy, vertex presentation, who did not experience premature rupture of membranes and delivered at gestational age ≥ 37 weeks.

P-values are based on chi-square analyses for categorical variables, independent samples Mann-Whitney U test for labor duration, and one-way ANOVA for the other continuous variables.

Similarly, duration of labor at home after contractions had become regular and ≤ 5-minutes apart among the later admits was not associated with the six study outcomes in the multivariable regression models (Table 4).

**Table 4:** Associations between labor duration at home after contractions had become regular and ≤ 5-minutes apart and the six study outcomes (n = 1,082).

Outcomes	Duration of labor at home after contractions became regular and ≤ 5-minutes apart	Adjusted OR	95% CI	p-value
Admitted in active labor (6–10 cm cervical dilation)				
	≤ 1 hour	Ref.		
	> 1–2 hours	1.00	0.67–1.50	0.995
	> 2 hours	0.92	0.62–1.36	0.679
Labor augmented via oxytocin				
	≤ 1 hour	Ref.		
	> 1–2 hours	1.02	0.75–1.40	0.881
	> 2 hours	1.14	0.85–1.53	0.392
Epidural analgesia				
	≤ 1 hour	Ref.		
	> 1–2 hours	0.90	0.60–1.33	0.588
	> 2 hours	0.71	0.49–1.02	0.063
Cesarean birth				
	≤ 1 hour	Ref.		
	> 1–2 hours	0.93	0.60–1.44	0.741
	> 2 hours	1.01	0.67–1.51	0.975
^a^Maternal morbidities				
	≤ 1 hour	Ref.		
	> 1–2 hours	1.28	0.91–1.81	0.158
	> 2 hours	1.19	0.85–1.65	0.313
^b^Neonatal morbidities				
	≤ 1 hour	Ref.		
	> 1–2 hours	1.50	0.95–2.37	0.080
	> 2 hours	1.00	0.62–1.60	0.996

Logistic regression models, controlling for maternal age, education, race/ethnicity, insurance coverage, high-risk pregnancy (Korst et al [36]), premature rupture of membranes, gestational age, and pain level before receipt of analgesia.

^a^Maternal morbidities: Composite measure indicating the occurrence of one or more maternal labor and delivery complications, identified by the ICD-9 CM codes reported in the hospital discharge data, as described in Korst et al [36].

^b^Neonatal morbidities: Composite measure indicating the occurrence of one or more neonatal labor and delivery related complications, identified by the ICD-9 CM codes reported in the hospital discharge data, as described in Korst et al [36].

## Discussion

In this prospective cohort study of nulliparous women with singleton pregnancies who began labor spontaneously, we found that the majority of women (65.3%) reported that their contractions had become regular and ≤ 5-minutes apart before hospital admission, and this factor was strongly associated with being in active labor on admission. Women whose contractions had become regular and ≤ 5-minutes apart before admission had labored at home 3 hours longer on average, and reported higher levels of pain before receipt of analgesia, in comparison to those whose contractions had not become regular and ≤ 5-minutes apart. Women who were admitted before their contractions had become regular and ≤ 5-minutes apart were different from those who were admitted after; they were more likely to have a high-risk pregnancy, premature rupture of membranes, and a gestational age < 37 weeks. However, 47.4% of the women who were admitted early had none of these conditions. Overall, women who were admitted before their contractions had become regular and ≤ 5-minutes apart were more likely to experience labor augmentation with oxytocin, epidural analgesia and cesarean birth. These results are consistent with previous studies which investigated outcomes of hospital admission in latent labor [1,9,44–46].

Surprisingly, we found no association between duration of labor at home after contractions had become regular and ≤ 5-minutes apart and the six study outcomes. Longer duration of labor at home after contractions had become regular and ≤ 5-minutes apart was not associated with increased cervical dilation on admission or decreased risk for labor augmentation with oxytocin, receipt of epidural analgesia or cesarean birth, as one might expect. These results do not provide support for common recommendations that women should continue to labor at home for an hour [31,33] or two hours [30,32] after their contractions have become regular and ≤ 5-minutes apart.

We found no differences between the early and later admission groups in the rate of maternal or neonatal labor and birth morbidities in either the bivariate analyses or the multivariable regression models. These results are similar to those reported in a study comparing women admitted in latent versus active labor, based on cervical dilation at admission [9]. Conversely, a study that compared women who labored at home ≤ 4 hours (early comers) to those who labored > 4 hours (late comers) reported higher rates of complications among the children of the women who were early comers, including hyperbilirubinemia and diagnosis of ‘difficult delivery’ [47]. However, our study was different from previous studies in that we defined early or later admission based on whether women’s contractions had become regular and ≤ 5-minutes apart before hospital admission.

We found that women who had attended childbirth education classes were more likely to labor at home until their contractions had become regular and ≤ 5-minutes apart. However, the majority of pregnant women in the US (58.5%) do not attend childbirth education classes [25]. A recent study using population-based data on American women’s experience of childbirth, found that women who attended childbirth education classes during pregnancy were less likely to experience primary cesarean birth and were more likely to attend a postpartum visit, to use birth control postpartum, to breastfeed and to adhere to safe infant sleep practices [25]. Childbirth education programs may also encourage participants to improve adherence to health promotion practices, such as cessation of smoking [48].

An important strength of this study is that we assessed factors related to women’s pre-hospitalization labor—including how long they had been in labor before admission, whether their contractions had become regular and ≤ 5-minutes apart before admission, and if so, how long they had labored at home after their contractions had become regular and ≤ 5-minutes apart. We found very little research which investigated associations between frequency of labor contractions on admission and intrapartum interventions, even though it is often recommended that women labor at home until their contractions have become regular and reached a specific frequency of occurrence. A limitation of this study was that we asked women about their labor and childbirth experience one month after birth. There was likely some level of recall bias. Additional limitations are that the women in this study were of higher socioeconomic status than the general population of women at first childbirth in the state of Pennsylvania [34], and women who were younger than 18 or older than 35 were not included. These factors may limit the generalizability of the study results.

While some guidelines suggest that admission of women in labor with uncomplicated pregnancies should be delayed until active labor has begun [49], the WHO cautions that further research is needed as to whether this should be a recommended policy [21]. In particular multi-center randomized controlled trials (RCTs) with adequate sample size are needed to investigate the risks and benefits of programs designed to delay hospital admission until active labor for women with uncomplicated pregnancies [13,14]. Because of the importance of frequency of labor contractions in recommendations as to when women in labor should present for hospital admission, future studies should measure the frequency of labor contractions more precisely than was possible in our study, as well as the duration of the contractions themselves, and investigate the association between these factors and labor and birth outcomes.

## Conclusions

As far as we know, this is the first study to investigate predictors and outcomes of hospital admission before or after labor contractions have become regular and ≤ 5-minutes apart. Our findings indicate that if a woman’s labor contractions have become regular and ≤ 5-minutes apart before hospital admission, she is more likely to be in active labor on admission, and less likely to experience the intrapartum interventions of labor augmentation with oxytocin, epidural analgesia and cesarean birth. However, we found no evidence to support recommending that women continue to labor at home for a specific time period after contractions have become regular and ≤ 5-minutes apart. These results provide information of potential use for childbirth educators and clinicians who care for women through the labor and delivery process.

## Supporting information

S1 Checklist STROBE statement—checklist of items that should be included in reports of *cohort studies*. (DOCX)
